# Oligomycin-producing *Streptomyces* sp. newly isolated from Swiss soils efficiently protect *Arabidopsis thaliana* against *Botrytis cinerea*

**DOI:** 10.1128/msphere.00667-23

**Published:** 2024-06-12

**Authors:** Fanny Louviot, Ola Abdelrahman, Eliane Abou-Mansour, Floriane L'Haridon, Pierre-Marie Allard, Laurent Falquet, Laure Weisskopf

**Affiliations:** 1 Department of Biology, University of Fribourg, Fribourg, Switzerland; 2 Swiss Institute of Bioinformatics, Lausanne, Switzerland; 3 Food Research and Innovation Centre, University of Fribourg, Fribourg, Switzerland; The University of Texas Medical Branch at Galveston, Galveston, Texas, USA

**Keywords:** *Botrytis cinerea*, *Streptomyces*, biocontrol, oligomycin, germicidin, antifungal activity, mycelial growth, spore germination, *Arabidopsis thaliana*

## Abstract

*Botrytis cinerea* is a necrotrophic phytopathogen able to attack more than 200 different plant species causing strong yield losses worldwide. Many synthetic fungicides have been developed to control this disease, resulting in the rise of fungicide-resistance *B. cinerea* strains. The aim of this study was to identify *Streptomyces* strains showing antagonistic activity against *B. cinerea* to contribute to plant protection in an environmentally friendly way. We isolated 15 Actinomycete strains from 9 different Swiss soils. The culture filtrates of three isolates showing antifungal activity inhibited spore germination and delayed mycelial growth of *B. cinerea*. Infection experiments showed that *Arabidopsis thaliana* plants were more resistant to this pathogen after leaf treatment with the *Streptomyces* filtrates. Bioassay-guided isolation of the active compounds revealed the presence of germicidins A and B as well as of oligomycins A, B, and E. While germicidins were mostly inactive, oligomycin B reduced the mycelial growth of *B. cinerea* significantly. Moreover, all three oligomycins inhibited this fungus’ spore germination, suggesting that these molecules might contribute to the *Streptomyces*’s ability to protect plants against infection by the broad host-pathogen *Botrytis cinerea*.

**IMPORTANCE:**

This study reports the isolation of new *Streptomyces* strains with strong plant-protective potential mediated by their production of specialized metabolites. Using the broad host range pathogenic fungus *Botrytis cinerea*, we demonstrate that the cell-free filtrate of selected *Streptomyces* isolates efficiently inhibits different developmental stages of the fungus, including mycelial growth and the epidemiologically relevant spore germination. Beyond *in vitro* experiments, the strains and their metabolites also efficiently protected plants against the disease caused by this pathogen. This work further identifies oligomycins as active compounds involved in the observed antifungal activity of the strains. This work shows that we can harness the natural ability of soil-borne microbes and of their metabolites to efficiently fight other microbes responsible for significant crop losses. This opens the way to the development of environmentally friendly health protection measures for crops of agronomical relevance, based on these newly isolated strains or their metabolic extracts containing oligomycins.

## INTRODUCTION

*Botrytis cinerea* is a fungal pathogen responsible for the disease known as “gray mold,” which affects over 200 mainly dicotyledonous plant species, causing major yield losses worldwide during both the cropping season and post-harvest storage (1). Current control measures heavily rely on repeated applications of chemical fungicides, leading to the development of *B. cinerea* resistance and posing threats to environmental and human health (2, 3). Therefore, there is a pressing need to find alternative ways to protect crops against this pathogen, such as the use of biocontrol agents. These agents could act by directly inhibiting various stages of the pathogen’s development cycle such as mycelial development, spore production or germination, and/or by stimulating the plant’s systemic defense mechanisms as reviewed by Poveda and colleagues (4).

Several microbial biocontrol agents have been reported to effectively control gray mold in different crops. For example, the yeasts *Exophiala jeanselmei* and *Cryptococcus albidus*, as well as *Erwinia* sp. (bacterium), and a non-identified coryneform bacterium showed effectiveness in roses (5), while the yeasts *Candida guilliermondii* and *C. oleophila* significantly controlled *B. cinerea* in tomato (6). In strawberries, various biological control agents against gray mold have been reported, including different yeasts (7), a variety of filamentous fungi (8, 9), as well as bacteria such as *Bacillus pumilus*, *B. subtilis*, *B. licheniformis*, *Pseudomonas fluorescens*, *Virgibacillus marismortui*, *Terribacillus halophilus*, *Halomonas elongata*, *Planococcus rifietoensis*, *Staphylococcus* spp., and two Enterobacteriaceae (7, 10, 11).

One bacterial genus, *Streptomyces*, has received considerable attention, and many studies have reported the potential of *Streptomyces* strains and their metabolites to control gray mold disease, such as *Streptomyces* sp. BS062 (12), *S. philanthi* RL‐1‐178, RM‐1‐138, and *S. mycarofaciens* SS‐2‐243 (13). Various metabolites have been identified as the causal agents of *B. cinerea* inhibition, including chloroxaloterpin A and B from *Streptomyces* sp. SN194 (14), actinomycin D from *Streptomyces* sp. sdu1201 (15), and oligomycin A from *Streptomyces* sp. FX13 (16). Moreover, the volatiles of *Streptomyces globisporus* JK-1 have been found to inhibit *B. cinerea* growth and development both on media and in inoculated tomato fruits (17), while the volatiles of *Streptomyces* sp. S97 inhibited the development of gray mold symptoms on strawberry fruits by more than 87% compared to unexposed control strawberries (18).

*Streptomycetes* are Gram-positive filamentous bacteria predominantly found in the soil (19). They are known to produce a wide range of natural products, accounting for approximately 70%–80% of commercially available antibiotics with pharmacological or agrochemical applications (20). While a plethora of studies have mined *Streptomyces* genomes and metabolomes to discover new antibiotic compounds of relevance for clinical application (21), the exploration of these particular soil microbial inhabitants’ potential to protect plant health is still in its infancy although their activity against fungal pathogens, including *B. cinerea,* has long been demonstrated, as mentioned earlier. In addition to their extensive production of specialized metabolites, *Streptomyces* have the ability to produce spores, which is a further asset when considering the tolerance to harsh conditions encountered in the plant phyllosphere (and, to a lesser extent, rhizosphere) these reproductive life forms offer, as well as the practical aspect of easier formulation and longer shelf life.

In view of all these promising features, this study’s two main aims were (i) to explore newly isolated soil-borne Actinomycetes for their ability to inhibit the development of *B. cinerea* both *in vitro* and *in planta* and (ii) to elucidate the chemical compound(s) mediating the observed pathogen inhibition.

## MATERIALS AND METHODS

### Soil samples and isolation of Actinomycetes

Nine soil samples were collected from Swiss soils randomly chosen, at the following locations (given in WGS84 coordinates): E: 6.62407/N: 46.84726; E: 7.15671/N: 46.79271; E: 6.47162/N: 46.75055; E: 6.60600/N: 46.84793; E: 6.63495/N: 46.79782; E: 6.63506/N: 46.79778; E: 6.64773/N: 46.84463; E: 6.63231/N: 46.79755; E: 7.15056/N: 46.79111. Ten grams of soil was suspended in 90 mL of sterile water; the solutions were then agitated at 210 rpm for 30 min and serially diluted to 10^−6^. One hundred microliters of these dilutions was spread on TSA medium (Tryptic Soy Agar for microbiology; Sigma-Aldrich) mixed with actidione (40 µg/mL) and nalidixic acid (10 µg/mL). The plates were then incubated at 28°C for 10 days, and 15 Actinomycetes were isolated based on their morphology.

### Molecular identification of the isolated strains by 16S sequencing and full-genome sequencing

Each Actinomycete strain was inoculated into 5 mL tryptic soy broth medium (Sigma-Aldrich, USA) and incubated at 28°C under shaking (180 rpm) for 3 days. Genomic DNA was extracted using the heat shock method (22). The 16S rDNA gene fragment was amplified via polymerase chain reaction (PCR) using the universal primers: 27F (5′-AGA GTT TGA TCC TGG CTC AG-3′) and 1492R (5′-CGG TTA CCT TGT TAC GAC TT-3′), which were purchased from Microsynth. PCR amplification and examination of the amplified PCR product through gel electrophoresis were conducted following the procedure outlined in Abdelrahman and colleagues (22). PCR products were then purified using the centrifugation protocol provided by the E.Z.NA. Cycle Pure Kit (Omega, bio-tek, USA). Each purified PCR product (12 µL) was combined with the forward primer (27F) (3 µL) and sent to Microsynth (Switzerland) for sequencing. The obtained sequences were compared with the available sequences in the database using nucleotide BLAST search in the NCBI GenBank database and submitted to GenBank (accession numbers: OR723505-OR723519). Subsequently, a phylogenetic tree was constructed using the Geneious software. The tree included the sequences of the 15 isolates and the sequences of their closest relatives obtained from the NCBI database. The tree construction employed the Tamura-Nei genetic distance model, a global alignment with free end gaps, and a similarity index of 65%, utilizing the neighbor-joining method.

To obtain full-genome information on the three selected strains S5.1, S11.8, and S13.2, their genomic DNA was extracted following the procedure outlined by Gillon and colleagues (23). Subsequently, the genomic DNA underwent processing by the next-generation sequencing (NGS) platform at the University of Bern according to the protocols and kits of Pacific Biosciences library preparation. The libraries were multiplexed on 1× SMRTcell 8M on a Sequel IIe. After sequencing HiFi long reads, the demultiplexing was performed with lima, and the HiFi reads were quality controlled with FastQC and assembled using the version 13.0.0.207600 of the SMRTlink pb_microbial_analysis pipeline. The polishing, circularization, and origin rotation of the assembly were provided by the SMRTlink pipeline if required. The genomes were annotated with Prokka (v. 1.13) (24) and antiSMASH (v. 7) (25). The sequences are available under project PRJEB74087, accession numbers ERR12778152, ERR12778246, and ERR12778259.

### Bacterial and fungal growth conditions and spore collection

Actinomycete strains were routinely grown on Bennett medium (1 g/L yeast extract, 1 g/L beef extract, 2 g/L peptone from casein, 10 g/L glucose, 15 g agar). *Botrytis cinerea* BMM (26) was routinely grown on potato dextrose agar (PDA) medium (Difco or Sigma-Aldrich). For *Streptomyces* spore collection, the strains were plated on ISP3 medium (27) and grown for 1 month at 28°C. The spores were then collected by flooding the Petri dishes with sterile distilled water and scratching the surface of the agar. The solution was filtered through glass wool to remove the mycelium and obtain pure spore solutions. For *Botrytis* spore collection, *B. cinerea* BMM was cultivated on PDA and the spores were harvested in a similar manner as described above for *Streptomyces*, with an additional centrifugation step (800 rpm for 10 min at 4°C) after glass wool filtration. The supernatant was then removed, and the pellet resuspended in sterile water.

### Preparation of *Streptomyces* cell-free filtrate

Five 7 mm plugs of *Streptomyces* colonies were transferred to 50 mL liquid Bennett medium, and this was agitated at 200 rpm, 28°C for 7 days. The liquid culture was then centrifuged for 20 min at 5,000 *g* and 4°C, and the pellet was removed. This step was repeated twice, and the solution was then filtered through a 0.22-µm filter to obtain cell-free filtrates (CFFs).

### Initial screening of the isolated Actinomycetes on different fungi

The 15 Actinomycetes soil isolates were initially tested against *Botrytis cinerea* BMM (26), *Rhizoctonia solani, Fusarium oxysporum* (both provided by Syngenta SA), and *Phytophthora brassicae* HH (28). After this initial screen, activity testing was repeated with replicates, this time replacing *P. brassicae* with the more agronomically relevant *P. infestans* (isolated by H. Krebs, Agroscope). To do so, 7 mm plugs of 1-week-old cultures of the different pathogens were placed in the center of a petri plate (PDA for *B. cinerea, R. solani,* and *F. oxysporum* and V8 for *P. infestans* [29]). Three 7 mm plugs of 1-week-old colonies of Actinomycetes were equidistantly placed at 10 mm from the border. The plates were then incubated at different temperatures depending on the ideal growth temperature of the respective pathogens (*Botrytis cinerea* at 19°C, *Rhizoctonia solani* at 17°C, *Fusarium oxysporum* at 28°C, and *Phytophthora infestans* at 17°C). For the control, a 7-mm plug of a non-inoculated plate of Bennet medium was used instead of bacterial plugs. Once the pathogens grown without bacteria (control) had reached the border of the plates, pictures were taken, and the area covered by the mycelium was quantified using ImageJ (30). This experiment was performed once with 3–7 technical replicates.

### *In vitro* effects of *Streptomyces* cell-free filtrate and volatiles on *Botrytis cinerea* growth and development

#### Effects on mycelial growth

Different concentrations (0.01%, 0.05%, 0.1%, 1%, 3%, 10%, 20%, 30%, 50%, and 60%) of CFFs were mixed with autoclaved PDA that had been cooled down to about 70°C and poured in Petri plates. A 7 mm plug from a 6-day-old culture of *Botrytis cinerea* mycelium was placed in the center of each plate. Sterile liquid Bennett medium was used as the control. The plates were incubated at 19°C, and the mycelial surface was measured after 5 days with ImageJ. The experiment was conducted once with three technical replicates.

To investigate the volatile-mediated activity of *Streptomyces* strains against *B. cinerea* mycelial growth, each strain was inoculated onto one compartment of a split plate filled with Bennett medium using five drops of 10 µL each of spore suspension (OD_600_ = 1). The plates were then preincubated at 28°C for three days. Subsequently, a 5 mm plug from a 4-day-old *B. cinerea* plate, or a 10 µL *B. cinerea* spore suspension (10^7^ spores/mL) was inoculated onto the centre of the other compartment. This setup only allowed for the exchange of volatiles between the two compartments (31). The plates were then sealed and incubated at 21°C for four days. Volatile-mediated antifungal activity was assessed by comparing the growth of the pathogenic fungus in plates containing the *Streptomyces* strains to that in control plates without the strains. The experiment was conducted once with five technical replicates, and measurements of the fungal growth area were performed using ImageJ software.

#### Effects on spore germination

Twenty-four-well microtiter plates were used and filled with 200 µL of 10 nM Tris-HCl (pH 7), 200 µL of potato dextrose broth (PDB) (Difco and Sigma-Aldrich), and 100 µL of spore suspension of *B. cinerea* at 10^4^ spores/mL. Different CFF volumes were then added to the wells (25, 50, 100, 250 and 500 µL), and the volume of the wells was adjusted to 1 mL with sterile distilled water. The plates were sealed and incubated at 19°C overnight in a shaker set at 100 rpm. Sterile liquid Bennett medium was used as the control instead of CFF. Spore germination and hyphal growth were observed with the Cytation 5 Cell Imaging Multi-Mode Reader (BioTek), for 8 days. To determine if the non-germinated spores were killed by the CFF, 100 µL of 10% propidium iodide was added to the wells containing 200 µL of 10 nM Tris-HCl (pH 7), 200 µL of PDB, 100 µL of a solution of *B. cinerea* spores at 10^4^ spores/mL, and 500 µL of CFF. The results were directly observed with the Cytation 5. A control was performed with Bennet medium instead of CFF. The experiment was conducted once with three technical replicates.

### *In planta* protection conferred by *Streptomyces* against *Botrytis* infection in *Arabidopsis*

#### Use of CFF as leaf spraying treatment

Eight-week-old *Arabidopsis thaliana* Columbia plants were dipped face down into CFF from each of the three selected *Streptomyces* strains at a concentration of 75% in Bennett liquid medium for 2 h and then air-dried for 2 h. Six leaves of each plant were then infected with 6 µL of *B. cinerea* spores diluted in ¼ PDB to 5 × 10^4^ spores/mL. The trays containing the plants were humidified, sealed with tape, and placed into the growth chamber (12 h light, 22.5°C, 12 h night, 19°C). The lesions were measured 3 days later. For each treatment, nine plants were used and distributed into three different trays. Sterile liquid Bennett (75%) was used as the negative control at the dipping stage (instead of CFF). Two independent experiments were performed.

#### Use of spores as soil-drenching treatment

The soils of 7-weeks-old Columbia plants (growing in pots with a diameter of 5.5 cm and a height of 4 cm) were inoculated with 5 mL of a spore suspension (6 × 10^4^ spores/mL in Bennett liquid medium) from each of the three selected *Streptomyces* strains. The plants were placed for one additional week in the growth chamber (12 h light, 22.5°C, 12 h night, 19°C), and then the infection and incubation were carried out as described above. For each treatment, nine plants were distributed into three different trays. Sterile distilled water was used as the control instead of the spore suspension. Two independent experiments were performed.

### Isolation and identification of *Streptomyces* active compounds

#### Chemicals and instruments

^1^H and ^13^C NMR spectra were recorded on a Bruker Avance 500 spectrometer (500 MHz) (Fällanden, Switzerland). The chemical shifts were referenced to tetramethylsilane (TMS). Electrospray mass spectrometry (ESIMS) HRESI-MS was performed on a Bruker FTMS 4.7T BioAPEX II. All solvents were HPLC grade (VWR, Nyon, Switzerland).

#### High-performance liquid chromatography

Sample analysis and purification were performed using an HPLC system (1260 Infinity, Agilent Technologies, CA, USA) with a quaternary pump, coupled to a diode array detector and a sample collector. The detection was fixed at 222 nm for oligomycin and 290 nm for germicidin. Two different HPLC conditions were developed. In condition A, for sample analysis, sample was injected on an MN 15/4.6 Nucleoshell RP18 2.7 µm column (Machery Nagel, Düren, Germany), eluted with a gradient of solvents of acetonitrile (ACN) and water, starting with 40% of ACN for 1 min and reaching 100% at 25 min for 3 min, with a flow rate of 0.4 mL/min. In condition B, for sample purification, sample was injected on a semi-preparative Ascentis C18 5 µm 15 cm × 10 mm column (Supelco, Bellefonte, PA, USA) eluted with a gradient of solvents ACN and water, starting at 60% ACN for 3 min, reaching 80% at 10 min, 98% at 15 min, and kept to 25 min, with a flow rate 1.2 mL/min.

#### Bioassay-guided isolation of the antifungal compounds

Five 7 mm plugs of one-week-old colonies of *Streptomyces* sp. strain S5.1 were transferred to 3 × 2 L canted Erlenmeyer containing 500 mL liquid Bennett medium and agitated at 200 rpm at 28°C for 7 days. The liquid culture was then centrifuged for 20 min at 5,000 *× g* and 4°C, and the pellet was removed. The filtrate (pH 4.5) was successively extracted with hexane, ethyl acetate, and methanol, and the solvents evaporated *in vacuo*, to obtain three extracts, hexane extract (HE; 28 mg), ethyl acetate extract (EAE; 315 mg), and methanol extract (ME; 141 mg). The ME resulted from the evaporation of the remaining liquid culture, which was dissolved in methanol. HE was fractionated by open column chromatography on silica gel 63–200, 60°A (Brunschwig, Basel, Switzerland) and eluted with gradient A (hexane/ethyl acetate from 100/1 to 1/100 [vol/vol]), which led to 10 fractions. Of these, fraction HE 8.2 (8 mg) was the most active one and yielded oligomycin B as a major compound with traces of oligomycin A and E, identified by HRESIMS. EAE was purified on a silica gel 63–200, 60 A open column, and eluted with gradient B (ethyl acetate/methanol from 90/10 to 0/100 [vol/vol]). The most active fraction, EAE 6 (8 mg), was purified on a SPE CHROMABOND C18 column, 45 µm, 3 mL/200 mg (Machery Nagel, Düren, Germany), eluted with hexane/ethyl acetate 2/8 (vol/vol), and led to the isolation of a mixture of compounds identified as germicidin A and B (4 mg) by HRESIMS and by comparison with standards (Chemical Cruz, TX, USA). Finally, for spore germination inhibition assay of *B. cinerea*, all fractions obtained during purification of HE and EAE were analyzed by HPLC with the gradient A described above. Fractions showing one or several identified compounds were combined and purified with HPLC on a semi-preparative column with gradient B to obtain germicidin B (3.1 mg), germicidin A (2.1 mg), oligomycin A (0.9 mg), oligomycin B (1 mg), and oligomycin E (0.7 mg).

### Antifungal activity of extracts, fractions, and pure compounds

#### Effects on *Botrytis cinerea* mycelium growth

The different extracts (HE, EAE, and ME) obtained from strain S5.1 CFF as well as all fractions resulting from their purification, as described above, were evaporated and dissolved in MeOH to a final concentration of 10 mg/mL. A volume corresponding to 10, 50, 100, 150, 200 µg was pipetted onto 6-mm Whatman discs and then let dry at room temperature. Two discs containing the same concentration of each fraction were positioned on either side of a PDA plate, and the control discs containing methanol were placed on the top. Four-day-old plugs (7 mm) of non-sporulating mycelium of *B. cinerea* were placed at the center of the plates. The control plate contained only the *B. cinerea* plug. The plates were incubated at 19°C with 12 h of light and 12 h of dark for 3 days, after which pictures were taken to measure the mycelial growth.

#### Effect of the purified compounds on *Botrytis cinerea* spore germination

Pure compounds of germicidin A and B and oligomycin A, B, and E were dissolved in ethanol to a final concentration of 0.6 mg/mL. Three dilutions of each compound were performed in PDB medium to obtain 5, 10 and 20 µg/mL. The control corresponded to ethanol diluted to 1/120, 1/60, and 1/30. In each well of a 96 well plate, a volume of 198 µL of each concentration was pipetted and mixed with 2 µL of spore solution at 10^6^ spores/mL in PDB. The plate was then incubated at 20°C with shaking at 115 rpm. The spore germination was observed after 7, 24, and 32 h with the Cytation 5. Three technical replicates were analyzed.

## RESULTS

### Identification of the isolated actinomycetes

**Fig 1:**
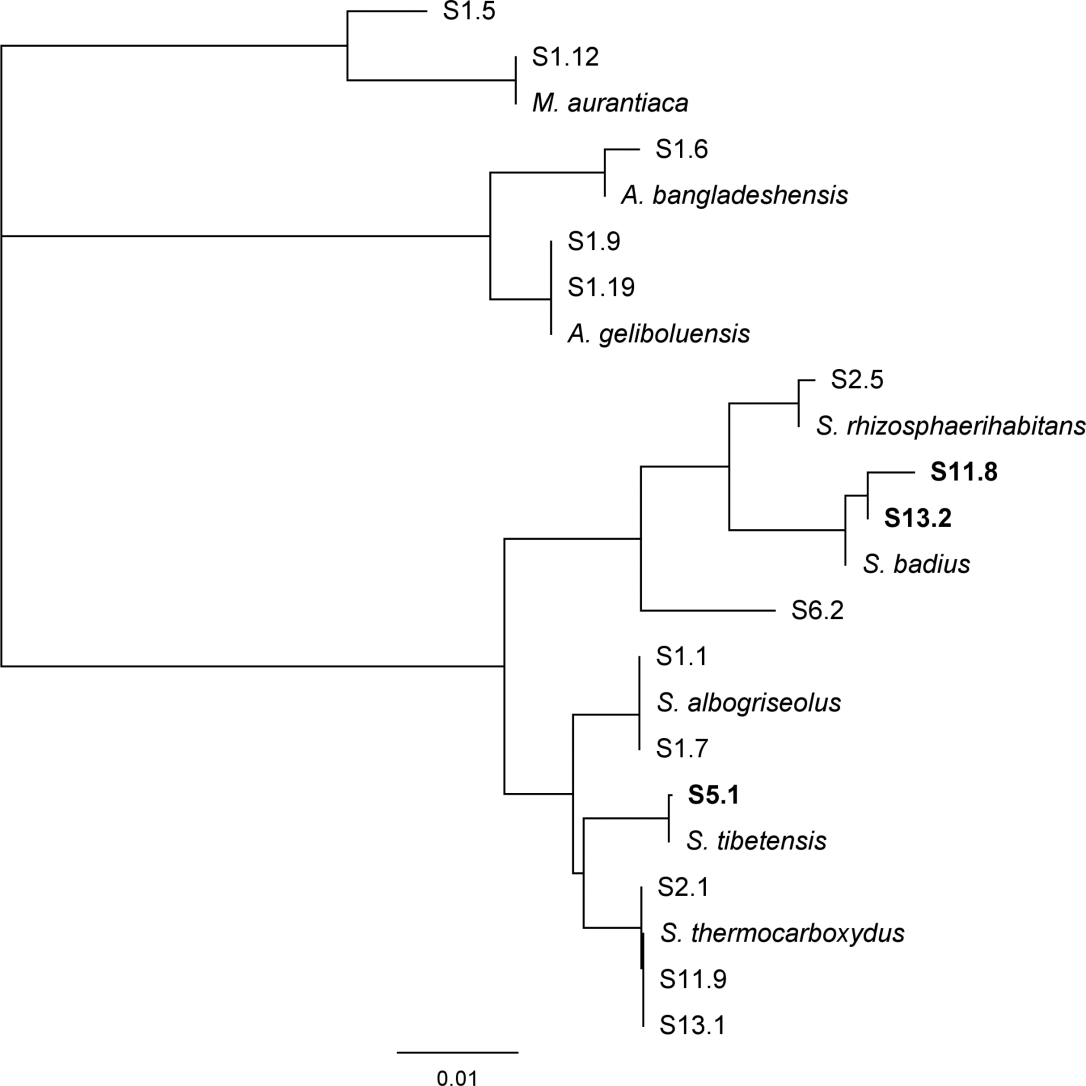
Phylogenetic tree of the isolated Actinomycetes. The tree was constructed using the Geneious software and was based on approximately 800 bp of the 16S rDNA gene sequence. Reference sequences from different species within the *Actinomadura* (***A***.), *Micromonospora* (***M***.), and *Streptomyces* (***S***.) genera, corresponding to the following accession numbers, were included: *Actinomadura bangladeshensis* (JQ972882.1), *Actinomadura geliboluensis* (MT490868.1), *Micromonospora aurantiaca* (LC551867.1), *Streptomyces albogriseolus* (MT229141.1), *Streptomyces badius* (MZ820169.1), *Streptomyces rhizosphaerihabitans* (OL414787.1), *Streptomyces thermocarboxydus* (MN704433.1), and *Streptomyces tibetensis* (NR_165779.1).

Fifteen bacterial strains belonging to the phylum *Actinobacteria* were isolated from nine soil samples randomly collected in Switzerland (Table S1). They were identified to the genus level by partial 16S rDNA sequencing, revealing that 10 strains belonged to the genus *Streptomyces* (S1.1, S1.7, S2.1, S2.5, S5.1, S6.2, S11.8, S11.9, S13.1, and S13.2), 3 to the genus *Actinomadura* (S1.6, S1.9, and S1.19), and 2 belonged to the genus *Micromonospora* (S1.5 and S1.12), as shown in the phylogenetic tree (Fig. 1).

### Screening for antifungal activity of the isolated Actinomycetes

**Fig 2:**
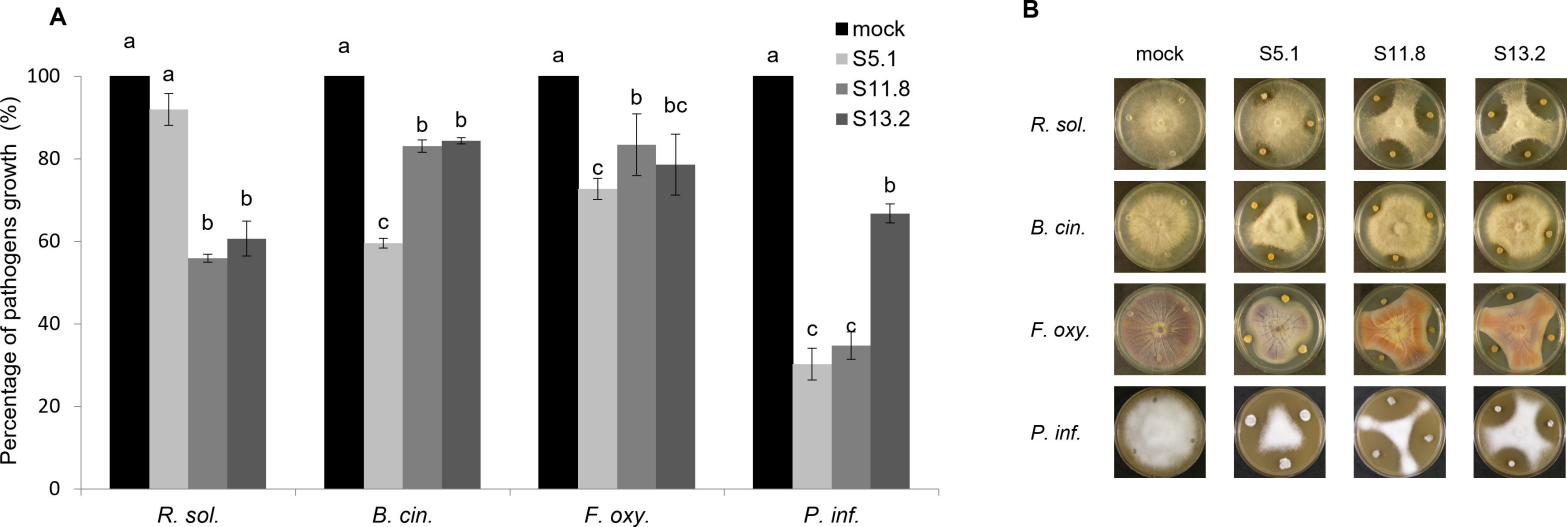
Co-cultivation assay showing the activity of strains S5.1, S11.8, and S13.2 against *R. solani, B. cinerea, F. oxysporum,* and *P. infestans*. (**A**) Quantification of the mycelial growth of the four tested pathogens. Bars represent the average of three technical replicates with standard errors. The percentage of pathogen growth was calculated by setting the growth in control plates to 100%. Significant differences according to a Student’s *t* test (*P* < 0.05, *n* = 3) are indicated by different letters. (**B**) Representative pictures of the dual assays with the different pathogens exposed to the *Streptomyces* strains or to the non-inoculated medium (mock).

A first screening of the 15 isolates for antifungal activity was performed with *Rhizoctonia solani*, *Botrytis cinerea*, *Fusarium oxysporum*, and *Phytophthora brassicae* as target pathogens, to gain an overview of the strains’ antagonistic potential (Table S1). This revealed three isolates of interest, all affiliated with the *Streptomyces* genus: S5.1, which, based on 16S sequence, clustered together with *S. tibetensis*, while S11.8 and S13.2, clustered together with *S. badius* (Fig. 1, bold font). Note that according to their full genomes, all three strains belong to new species, with S11.8 and S13.2 belonging to the same new species and S5.1 to a different one. After this initial screen, we repeated the antifungal activity assay with these three strains, this time replacing *P. brassicae* with the agronomically more relevant *P. infestans*. This confirmed the initial screen and revealed that all three strains were able to significantly inhibit *B. cinerea, F. oxysporum,* and *P. infestans,* while only S11.8 and S13.2 also significantly inhibited the growth of *R. solani* (Fig. 2).

As *Streptomyces* are known to have the capacity to emit bioactive volatile compounds, we tested whether part of the observed inhibition of *B. cinerea,* which we decided to focus on for the rest of the study in view of its importance as broad-host pathogen, could also be linked to the emission of volatiles by carrying out a split plate assay. This revealed that the inhibiting effect of the strains on *B. cinerea* mycelial growth was almost exclusively due to diffusible (non-volatile) compounds, as no inhibition was observed when the fungus was inoculated as a mycelial plug (Fig. S1). When it was inoculated as spore suspension, varying but rather modest inhibition was observed, mainly for S5.1, suggesting the emission of volatile compounds inhibiting spore germination by this strain (Fig. S1).

### Culture filtrates from the three isolates recapitulate the strains’ effects on *Botrytis cinerea* mycelial growth

**Fig 3:**
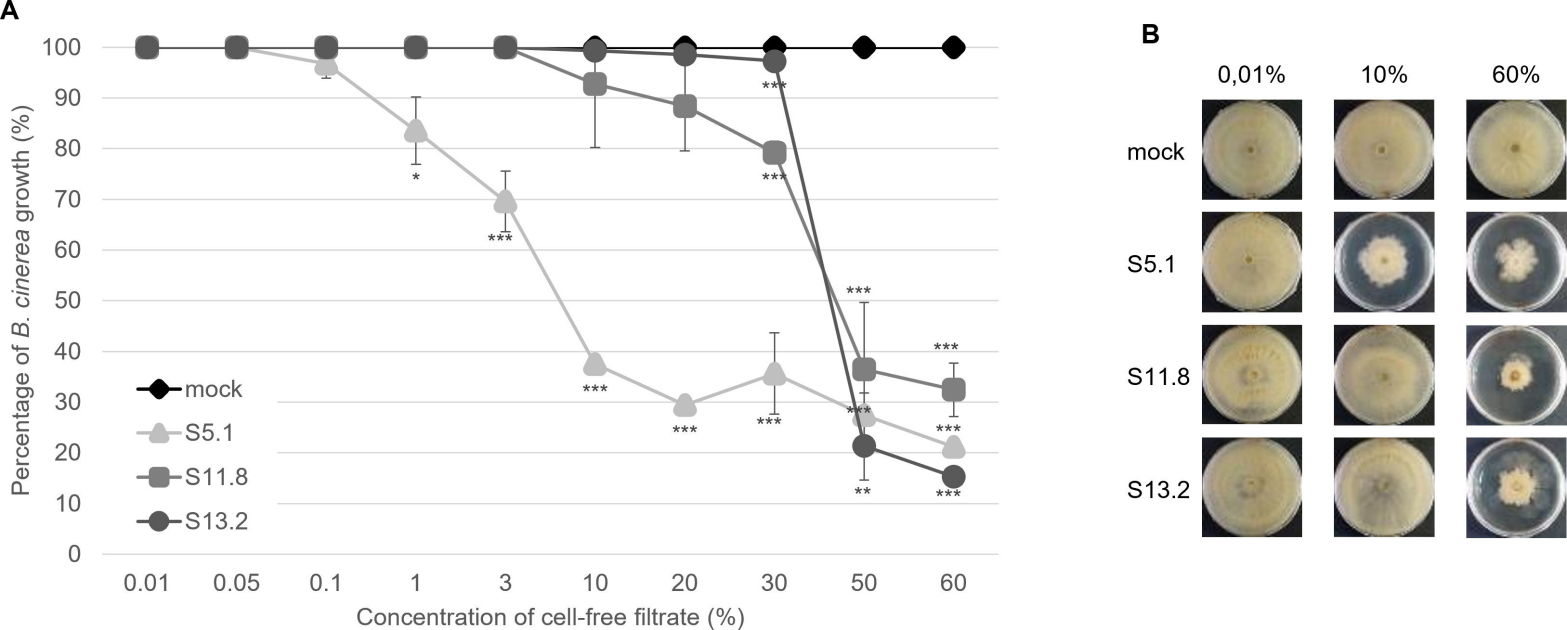
*Botrytis cinerea*’s growth on PDA mixed with different concentrations of cell-free filtrates from the strains S5.1, S11.8, and S13.2. (**A**) Quantification of *B. cinerea* mycelial growth. Each point represents the average of three technical replicates with standard errors. Percentage indicates the petri dish coverage (with 100% meaning that the petri dish was fully covered). Significant differences according to a Student’s *t* test are indicated by asterisks (*P < 0.05; **P < 0.01; ***P < 0.001, *n* = 3). This experiment was repeated twice with very similar results. (**B**) Representative pictures of *B. cinerea* mycelial growth after 5 days in the medium containing the strains’ CFF or in non-inoculated medium (mock).

Once the effect of volatiles could be ruled out, we wanted to assess whether the observed pathogen mycelial growth inhibition was mediated by secreted chemical compounds or enzymes rather than by direct cell-cell interactions. We, therefore, tested the effect of CFFs on the mycelial growth of *Botrytis cinerea*. Mixing different concentrations of the CFF of strains S5.1, S11.8, and S13.2 into the medium prior inoculation of *B. cinerea* revealed a similar activity profile as that observed for the strains: the CFF from strain S5.1 was the most active, with a decreased mycelial growth observed already with a concentration of 1% in PDA medium (IC_50_ of ca. 7%), while the CFF of strains S11.8 and S13.2 started to cause a significant inhibition only from higher concentrations on and had a higher IC_50_ (ca. 40%) (Fig. 3).

### *Botrytis cinerea* spore germination is also affected by the stains’ cell-free filtrates

**Fig 4:**
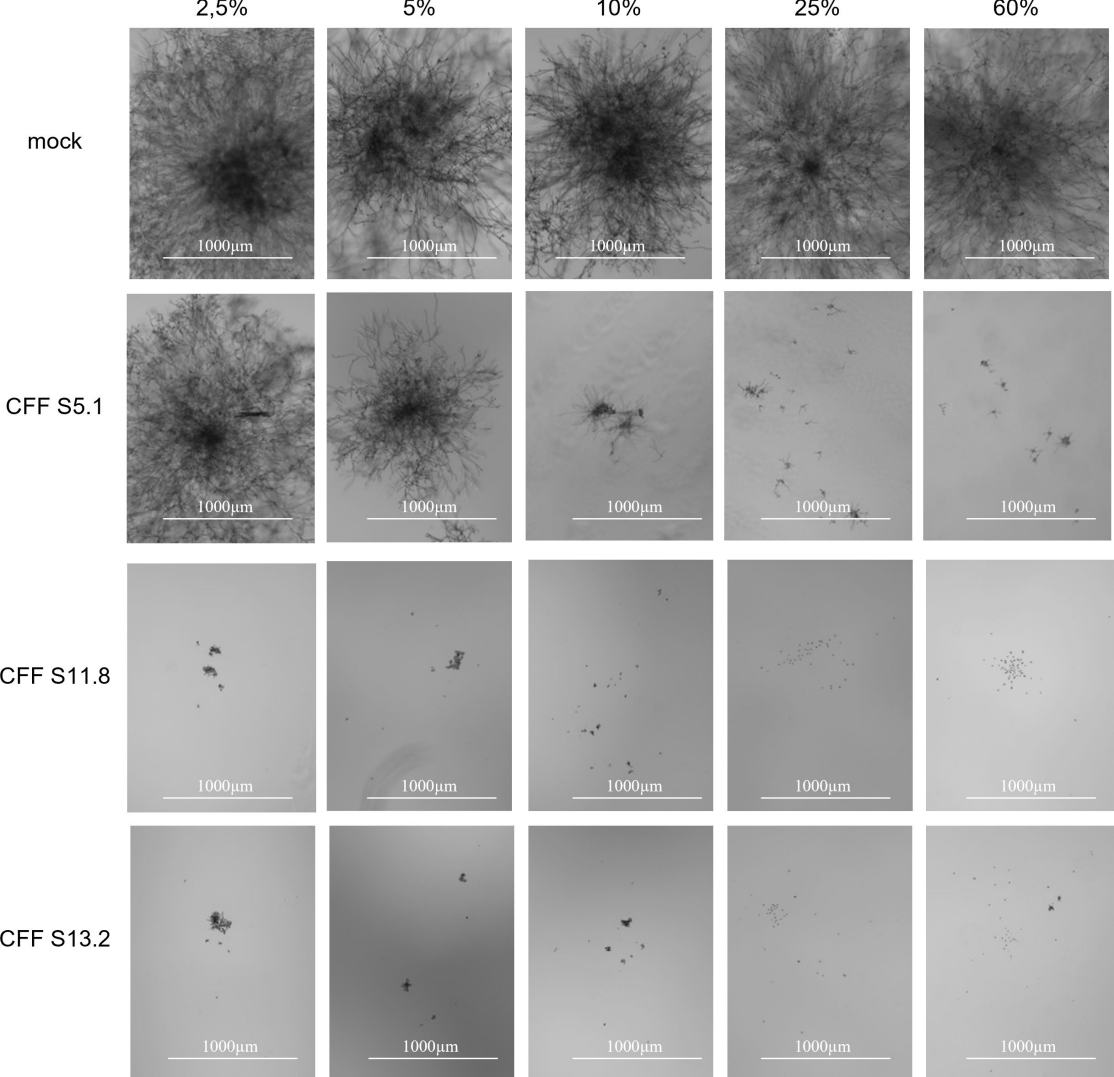
Dose-dependent activity of *Streptomyces*’s CFF on *Botrytis cinerea* spore germination. Spore germination of *Botrytis cinerea* incubated in media containing different concentrations of CFF from strains S5.1, S11.8, and S13.2 is visualized after 2 days. Pictures were taken with the Cytation 5 Cell Imaging Reader from Biotek at a 4-fold magnification.

To assess whether next to mycelial growth, the epidemiologically important process of spore germination would also be affected by the *Streptomyces*’s CFF, we followed *B. cinerea* spore germination in media containing different CFF concentrations (from 2.5% to 60%). We observed a dose-dependent activity of the CFF of the three *Streptomyces* strains, with the highest activities for strains S11.8 and S13.2 (Fig. 4). After 2 days of incubation with at least 10% CFF from both strains, germination was completely suppressed. Strain S5.1’s CFF did not lead to complete inhibition of germination even at the highest tested concentration. However, germination was also strongly delayed when this strain’s CFF was added at a concentration of at least 10% (Fig. 4).

As no germination was observed 2 days after inoculation with high concentrations of the CFF from strains S11.8 and S13.2, the experiment was extended to 8 days to check whether this inhibition would hold in the long term and if it would, whether the spores would still be alive or dead. After 8 days, as no spore germination was observed for spores incubated in a solution containing ≥10% of CFF from strains S11.8 and S13.2, a propidium iodide stain was carried out on these samples, which revealed that most of these spores were dead (Fig. S2).

### The *Streptomyces* CFF confer protection against *B. cinerea* infection in the model plant *Arabidopsis thaliana*

**Fig 5:**
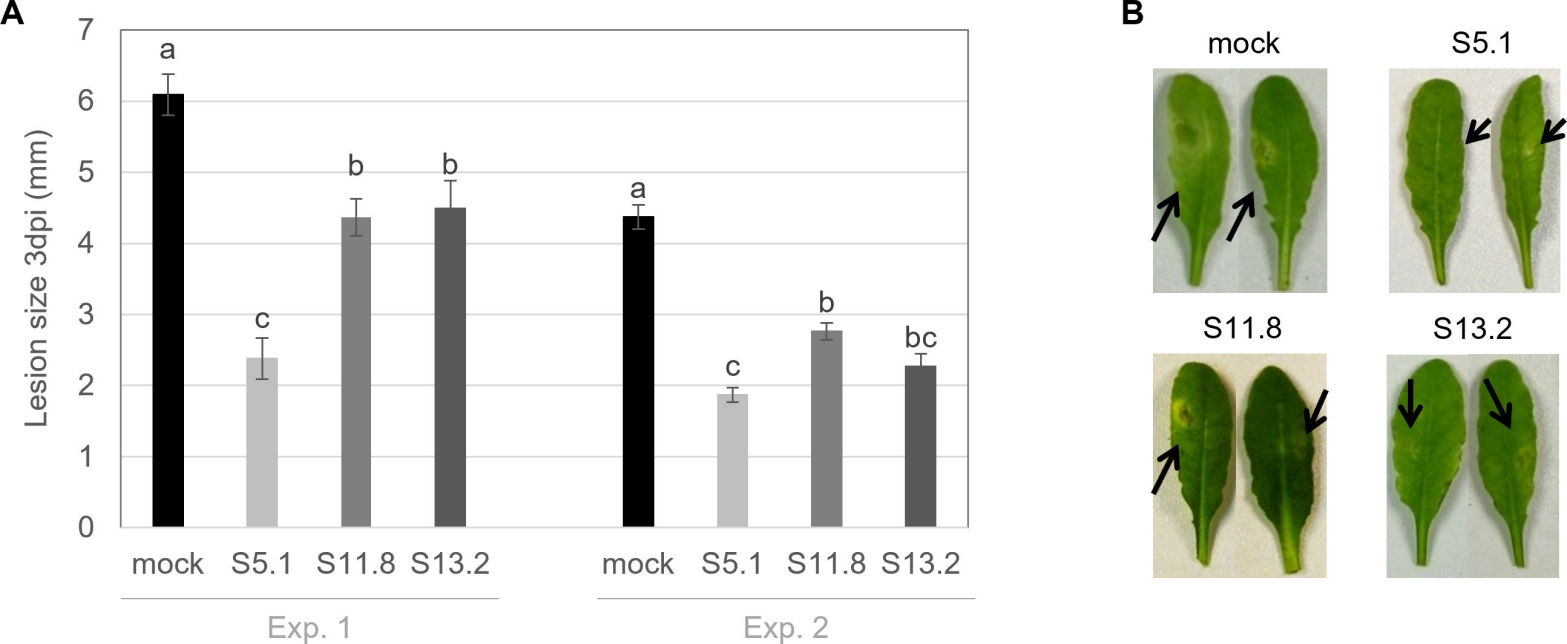
Reduction of infection symptoms in plants treated with the *Streptomyces*’s CFF. (**A**) The size of the lesions following *B. cinerea* infection in *A. thaliana* plants was measured 3 days post infection in two independent experiments (Exp. 1 and Exp. 2). Plants were either dipped into the CFF of strains S5.1, S11.8, or S13.2, or dipped into liquid Bennett medium in the mock treatment. Bars represent the averages of lesion sizes from 54 infected leaves from nine different plants separated in three independent groups. Statistical analyses indicated that there was no batch effect, allowing us to pool the data within each experiment. Significant differences between treatments according to a Student’s *t* test (*P* < 0.05, *n* = 54) are indicated by different letters. Arrows are pointing to the lesions. (**B**) Representative pictures of leaves infected with *B. cinerea* after being treated with CFF of S5.1, S11.8, S13.2 or with mock.

In view of the dual effect of the three strains’ CFF on both mycelial growth and spore germination of *B. cinerea*, we wondered whether the molecules contained in the culture extracts would also be able to inhibit the pathogen progression *in planta*. We, therefore, carried out leaf infections with *Botrytis cinerea* in plants previously treated with each strain’s CFF and compared the disease progress on these treated plants compared with the mock-treated plants. Although plants had been dipped into undiluted CFF prior to the infection with *B. cinerea* spores, we observed no phytotoxicity of the treatments. All strains’ CFF led to a significant reduction of the lesion sizes, in two independent experiments (Fig. 5). Interestingly, strain S5.1’s CFF, which was less efficient than the others in the spore germination assay (Fig. 4), proved to be the most efficient one in this plant infection assay, corroborating the observation that this strain’s CFF also caused the strongest mycelial growth inhibition (Fig. 3).

### Identification and structure determination of active compounds revealed oligomycins, but not germicidins, as responsible molecules for the anti-*Botrytis* activity

In order to identify the active compound(s) responsible for *B. cinerea* inhibition, the CFFs of the three strains were compared by HPLC (Fig. S3A and B). This revealed two families of compounds, as indicated by their UV spectra, which were found in the most active strain S5.1 but not in strains S11.8 and S13.2. For compound identification, the CFF of strain S5.1 was successively extracted with hexane, ethyl acetate, and methanol, and the resulting extracts were tested against mycelial growth of *B. cinerea*. In this disc diffusion assay, the hexane extract showed the highest inhibition, followed by the ethyl acetate extract, while hardly any inhibition was observed for the methanol extract (Fig. S4). Both the hexane and the ethyl acetate extracts were subjected to further bio-assay guided purification.

**Fig 6:**
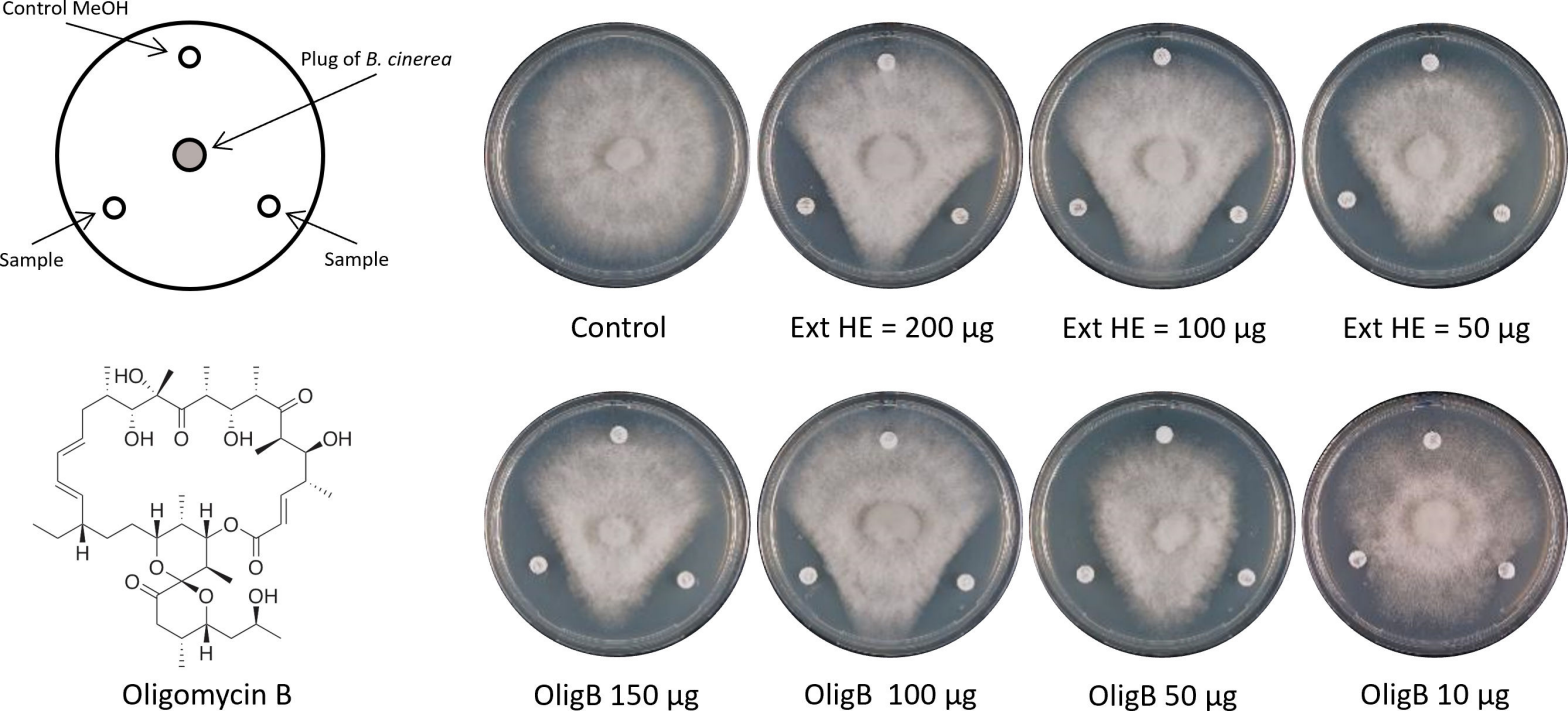
Activity of purified oligomycin B on *Botrytis cinerea* mycelial growth. Antagonism effect of oligomycin B and of the hexane extract at different concentrations (50, 100, and 200 µg for the hexane extract and 10, 50, 100, 150 µg for oligomycin B) on the mycelial growth of *B. cinerea*. The respective quantity of each compound dissolved in methanol was deposited on Whatman discs and let dry before to test their activity on PDA medium against the mycelial growth of *B. cinerea*. The pictures were taken when the mycelium of *B. cinerea* had invaded the entire control plate, i.e., after ca 72 h incubation at 19°C with 12 h of light/12 h of darkness. The experiment was performed once.

The hexane extract led to the identification of oligomycin B (8 mg) as a major active compound together with traces of oligomycin A (0.9 mg) and E (0.8 mg). The structure of oligomycin B was deduced on the basis of HRESIMS as *m/z* 827.4915 [M + Na]^+^, calcd. 827.4921 for C_45_H_72_O_12_Na, and by ^1^H and ^13^C NMR (32). The structures of oligomycin A *m/z* 813.5111 [M + Na]^+^ C_45_H_74_O_11_Na and oligomycin E *m/z* 843.4865 [M + Na]^+^ C_45_H_72_O_13_Na were deduced by comparison of the fragmentation pattern of HRESIMS/MS with oligomycin B spectra (Fig. S5 and S6; Table S2), which were in accordance with the reported fragmentation pattern for oligomycin A (33). Both the entire hexane extract and the fraction 8.2 containing the major compound oligomycin B were tested against *B. cinerea* mycelial growth at concentrations ranging from 50 to 200 µg for the hexane extract and from 10 to 150 µg for the purified oligomycin B (Fig. 6). The minimum concentration required for the inhibitory activity of oligomycin B against *B. cinerea* mycelial growth was 10 µg per disc.

**Fig 7:**
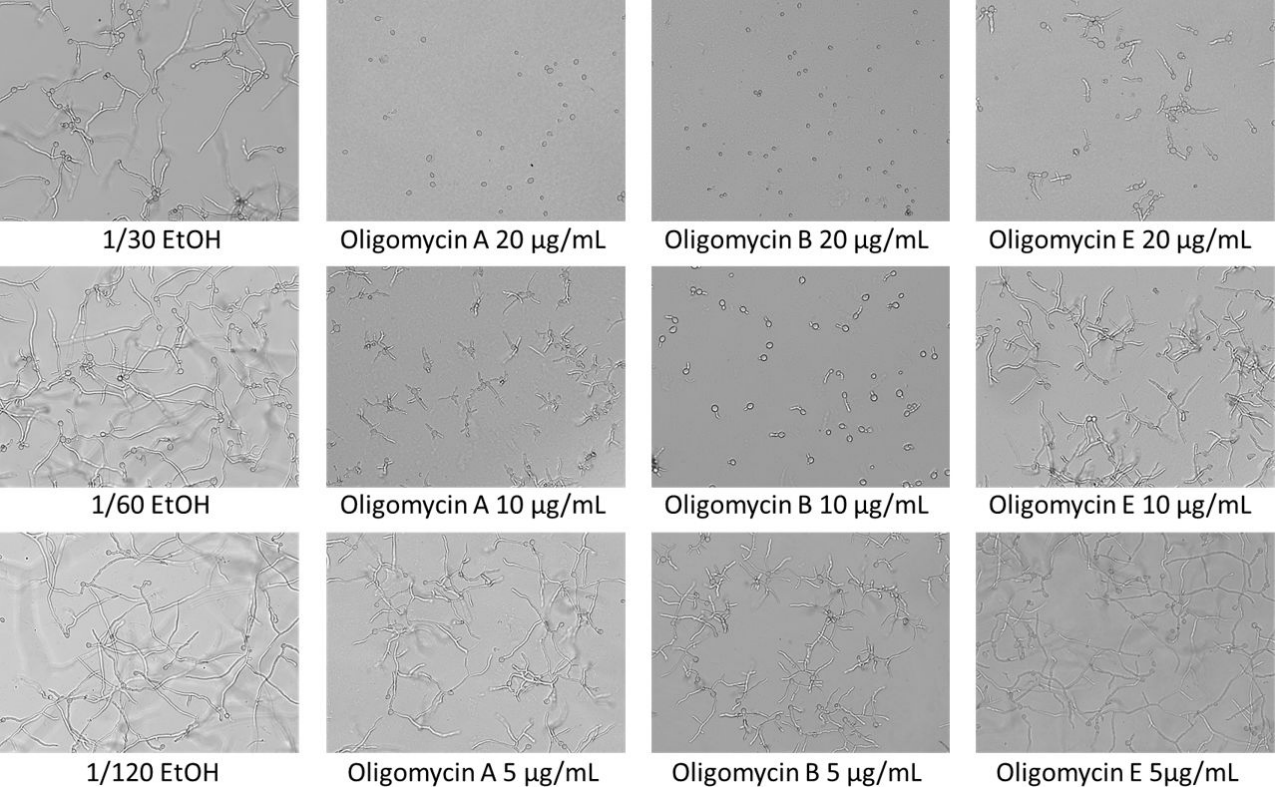
Activity of purified oligomycin A, B, and E on the germination of *B. cinerea* spores. The effect of oligomycin A, B, and E on the germination of *B. cinerea* spores was assessed at different concentrations (20, 10, and 5 µg/mL). Each purified oligomycin was solubilized in ethanol to obtain a concentration of 0.6 mg/mL and thereafter diluted in PDB to obtain the three final concentrations tested. Ethanol diluted in PDB was the control. In each well, 198 µL of each solution was mixed with 2 µL of *B. cinerea* spores at a concentration of 1 × 10^6^ spores/mL. After incubation of 24 h at 20°C, pictures were taken with the Cytation 5 Cell imaging reader from Biotek at a 10-fold magnification. For each treatment, three replicates were analyzed.

Since the CFF of strain S5.1 also led to delayed spore germination, we tested the activity of oligomycins on spore germination (Fig. 7). This revealed that oligomycin B, which was the most abundant and most active molecule of the family, strongly delayed spore germination at a concentration of 10 µg/mL, while it totally suppressed it when applied at a concentration of 20 µg/mL. Oligomycin A was the next most active compound, followed by oligomycin E (Fig. 7).

Since some anti-*Botrytis* activity was also detected in the ethyl acetate extract, this extract was also analyzed and led to the identification of germicidin A *m/z* 197.1177 [M + H]^+^, C_10_H_14_O_3_ and germicidin B *m/z* 183.1014 [M + H]^+^, C_11_H_16_O_3_. The structure of germicidins was determined by HRESIMS and compared by HPLC with the commercial standard. Germicidin A and B were tested as equal amounts of each compound in the same fraction up to a concentration of 100 µg, but neither activity on mycelial growth (Fig. S7) nor on spore germination (Fig. S8) of *B. cinerea* was observed. This suggests that oligomycins, but not germicidins, were at least partly responsible for the observed inhibition of both mycelial growth and spore germination by the CFF of strain S5.1.

### Candidate genes and compounds possibly underlying the activity of strains S11.8 and S13.2

While the CFF of S5.1 was more efficiently inhibiting *B. cinerea* mycelial growth and showed highest plant protection against this pathogen, the CFFs of S11.8 and S13.2 induced stronger inhibition of spore germination. Analyzing the full genomes of the three strains confirmed that S11.8 and S13.2, indeed, did not harbor the oligomycin biosynthetic gene cluster. However, antiSMASH analysis also revealed that the two strains shared 15 biosynthetic gene clusters which were absent from S5.1, among which genes encoding siderophores, polyketides, and a phenazine, which all could potentially account for their activity on spore germination (Table S3). Out of these, we could detect the siderophore EDHA (ethylenediaminesuccinic acid hydroxyarginine) and the endophenazine B in the cell free filtrates of the strains, which shows that they were not only encoded in the genome but also produced in the culture conditions used to assess the biological activity of the CFFs (Table S4).

## DISCUSSION

This study’s first aim was to isolate new Actinomycete strains with plant protection potential from different soils in Switzerland. We, indeed, could isolate Actinomycetes from the nine soils investigated, which reflects these bacteria’s prevalence in the soil environment (34, 35) even if the selected soils represented diverse ecosystems, going from forests to lakeshores over botanical and vegetable gardens (Table S1). Among the 15 isolates tested, three strains showed an inhibitory activity against a range of fungal and oomycete pathogens (Table S1). All three belonged to the *Streptomyces* genus, which is the most prolific antibiotic producing genus among the phylum *Actinobacteria* (20, 36, 37). From the three strains, S5.1 strongly inhibited the mycelial growth of the two tested Ascomycetes (*B. cinerea* and *F. oxysporum*) as well as the oomycete *P. infestans* but was inefficient against the Basidiomycete *R. solani*. S11.8 and S13.2 had significant inhibitory effects on all four pathogens, they were therefore much more active than S5.1 on *R. solani,* but much less on *B. cinerea* (Fig. 2). This differential activity matched the different phylogenetic relationship between the three strains, S11.8 and S13.2 clustering together with *Streptomyces badius,* while S5.1 belonged to a different branch within the *Streptomyces* isolates and was most closely related to *S. tibentensis* (Fig. 1). To the best of our knowledge, strains from the newly described Actinomycete *S. tibetensis* species (38) have not yet been reported to exhibit any biological activity. In contrast, *S. badius* is known for producing various specialized metabolites with biological activity. For instance, dinactin has shown antagonistic activity against the rubber anthracnose fungus *Colletotrichum gloeosporioides* by inhibiting the production and germination of its spores (39). Additionally, hydroxy marilone C, produced by *S. badius*, displayed potent antioxidant, antitumor, and antiviral activities (40). Another strain of *S. badius* isolated from a Sahara soil exhibited considerable antagonistic activities against *Fusarium culmorum*, *Verticillium dahliae,* and *Fusarium oxysporum f.* sp. *albedinis* (41). Furthermore, *S. badius* was also isolated from the intestinal microbiota of Colorado potato beetles’ larvae (*Leptinotarsa decemlineata*) and demonstrated inhibitory activity against multiple Gram-positive bacteria, *Aspergillus niger*, and *Saccharomyces cerevisiae* (42). All these documented biological activities of *S. badius* corroborate the inhibitory potential observed with our strains, specifically S11.8 and S13.2, against multiple fungal pathogens.

Further experiments focused on *B. cinerea* in view of its very large plant host range (1). They showed that the mycelial growth inhibition observed in the dual culture plates was likely due to metabolites or other secreted molecules. Indeed, a similar gradient of activity was visible with the strains’ CFF as that observed with the strains themselves, S5.1 being more active than S11.8 and S13.2 (Fig. 3). This difference between S5.1 and the two other strains was also visible in an *in planta* protection assay we performed in the model plant *Arabidopsis thaliana*, especially in the first repetition (Fig. 5). We next wondered which metabolites could be responsible for such an inhibitory effect of S5.1. HPLC analysis revealed both germicidin and oligomycin as present only in S5.1 but absent from S11.8 and S13.2, making these compounds putative candidates to explain the higher inhibitory activity of S5.1 (Fig. S4). The γ-pyranone pyrolactone compounds germicidin A and B are known as autoregulative inhibitors of spore germination in the genus *Streptomyces* (43). However, when tested as pure equal mixture of germicidin A and B on *B. cinerea* mycelial growth, germicidins did not show any activity (Fig. S6) and, therefore, likely do not account for strain S5.1’s particular anti-*B*. *cinerea* activity (Fig. 1 to 3). On the other hand, when we performed a bioassay-guided fractionation of strain S5.1 culture, we identified oligomycin B as the major compound (76%) together with traces of oligomycin E (12%) and oligomycin A (10%) in the active fractions (Fig. S5; Table S2). Oligomycins are 26-membered macrolides with a spiroketal moiety. Ōmura and colleagues (44) identified the biosynthetic gene cluster encoding the polyketide backbone of oligomycin in *S. avermitilis*, yet members of the oligomycin family have been isolated from different *Streptomyces* spp. (45): Oligomycin A was the first member of the family to be isolated from a soil *Streptomyces* belonging to the species *S. diastatochromogenes* as antifungal compound active against *Blastomyces dermatitidis* (46). Oligomycin B was identified later from the same strain culture that had led to the identification of oligomycin A (47). Oligomycin E was later reported to be produced by a *Streptomyces* sp. strain isolated from soil in Tasmania and to show antibacterial and antitumoral activity (48). Oligomycin A and B, on the other hand, have been shown to inhibit the ATP synthase (49, 50). An early study investigated the biological activity of the three oligomycins A, B, and E against 40 different fungi, most of which were inhibited by these molecules. Among them a *Botrytis* sp. strain was inhibited by oligomycins A and B at a minimal concentration of 5 µg/mL after 2 days, while 30 µg/mL was needed to still observe an inhibition after ten days (51). Beyond fungi, oligomycin A and B were reported to impair motility and cause subsequent lysis of *Plasmopora viticola* zoospores in a dose- and time-dependent manner. Oligomycin B showed higher motility inhibitory and lytic activities (IC_50_ 0.15 and 0.2 µg/mL) than oligomycin A (IC_50_ 3.0 and 10.0 µg/mL) (52).

In the present study, we also observed that oligomycin B was inhibiting the mycelial growth of *B. cinerea,* with a minimum inhibition concentration of 10 µg in the disc diffusion method (Fig. 6). The activity threshold obtained with the disc diffusion method (this study) and with the agar diffusion method [the study of Marty (51)] cannot be directly compared, but in any case, we can safely say that this molecule likely participated to the inhibitory activity of S5.1 observed in the mycelial growth of *B. cinerea* (Fig. 3) and potentially also in the *in planta* protection conferred by this strain (Fig. 5). One additional and important developmental stage of the pathogen is the germination of spores, which was reported as particularly sensitive to inhibition (53). When testing the effect of the three oligomycins on *B. cinerea* spore germination, we noticed that all three significantly delayed or even totally prevented spore germination, depending on the concentration used. Oligomycin B, which was most abundant in strain S5.1’s extracts, was also the most active compound, followed closely by oligomycin A and, to a lesser extent, by oligomycin E (Fig. 7). These observations are in line with a recent study reporting inhibitory activity of oligomycin A on both spore germination and germ tube elongation in *B. cinerea* (16). Although the anti-*Botrytis* activity of oligomycin B was clearly demonstrated in our study, the use of this pure compound for crop protection would not be recommended, as its molecular target (ATP-ase) is widespread and conserved among organisms; therefore, unwanted effects on non-target organisms would be expected. However, when produced locally and in low concentrations, in combination with other active molecules, this compound likely contributes to the overall positive action of biocontrol agents producing it.

Interestingly, the CFF of strains S11.8 and S13.2, in which no oligomycins were detected, were even more efficient inhibitors of spore germination in our experiment than that of strain S5.1 (Fig. 4), suggesting that other compounds beyond oligomycins account for the CFF’s overall activity and that this activity cannot be restricted to a single molecule or molecule family. Beyond the need to consider more than one molecule when attempting to explain the antagonistic activity of a strain’s CFF, we also need to consider that more than one mode of action will be involved in the ability of the strains themselves to act as biocontrol agents.

In line with this hypothesis, we observed that all three strains significantly protected the plant against infection not only when their CFF was applied to the leaves (Fig. 5), but also when we treated the soil in which the plants grew with spores from the three strains, prior to infecting the leaves with *B. cinerea*. The results showed that all three strains were significantly protecting the plants remotely, and that in this setup, strain S11.8 was as effective a protector as strain S5.1, with strain S13.2 being slightly less active than S5.1 but similar to S11.8 (Fig. S9). In such an experimental setup where the biocontrol agents (soil-inoculated) and the pathogen (leaf-inoculated) are physically separated, the most likely mode of protection is the induction of resistance in the plant by the bacteria. Indeed, plant beneficial microorganisms are known to stimulate plant immunity through the so-called induced systemic resistance (ISR), which confers protection against a wide range of pathogens (54). Although we cannot exclude that a soil-inoculated strain—or a compound secreted at the root level—could in principle migrate to the leaves where the infection took place and act directly on the pathogen there, the relatively short time between soil inoculation and leaf infection (1 week) argues for ISR rather than for a direct inhibiting effect of either the strains or their metabolites. To date, the induction of systemic resistance by *Streptomyces* species in plants has been well documented. For instance, *S. chromofuscus* strain RFS-23 has been found to stimulate systemic resistance in tomato plants and activate their defense responses against yellow leaf curl virus infection (55). Abbasi and colleagues (56) also observed that different *Streptomyces* strains induce resistance to Fusarium wilt in tomatoes through distinct molecular mechanisms suggesting strain specific-resistance induction. Furthermore, the efficacy of *Streptomyces* strains in enhancing rice plant defense has been demonstrated against bacterial leaf streak disease caused by *Xanthomonas oryzae* pv. *oryzicola*, whether treating the plant seeds with single *Streptomyces* strains or with a consortium of two *Streptomyces* strains (57). Additionally, a *Streptomyces corchorusii* strain and a *Streptomyces misionensis* strain were shown to suppress the bacterial panicle blight disease caused by *Burkholderia glumae* in rice plants through upregulating plant defense gene expression (58).

In conclusion, this study which initiated from an attempt to isolate new candidate biocontrol agents led to the discovery of three promising strains showing differential activities on different pathogens, and differential modes of action on the model pathogen selected here*—Botrytis cinerea*. Furthermore, oligomycin B was highlighted as a candidate molecule explaining at least partially the inhibition of both mycelial growth and spore germination observed for strain S5.1, while germicidins, which was also exclusively present in this strain, were not active on *B. cinerea* (Fig. S6 and S7). The three strains appear as promising candidates for biocontrol, one of them being particularly efficient at the mycelial growth stage, the two others at the spore germination stage, and the three offering significant *in planta* protection against *Botrytis* infection either via their metabolites sprayed on the leaves or via their inoculation in the soil. In view of their good performance as single strains but their varying metabolome and capacity to inhibit different stages of the pathogen life cycle, it would seem worthwhile to try and combine the three strains, to take advantage of their particular abilities and ideally reach a synergetic protective effect that could be harnessed both through soil inoculation and leaf treatment (59). However, before further steps toward application can be taken, many open questions remain to be solved, the most important of which would be whether the protective effects observed on the model plant *Arabidopsis* would also be achieved when *Botrytis* would grow on other, agronomically relevant host plants.

## SUPPLEMENTAL MATERIAL

Supplemental figures msphere.00667-23-s0001.docx Figures S1-S9.

Table S1 msphere.00667-23-s0002.xlsx Isolation, identifcation, and activity of the isolated strains against the mycelial growth of multiple pathogens.

Table S2 msphere.00667-23-s0003.xlsx ESI-MS/MS fragmentation patterns of oligomycins A, B, and E,

Table S3 msphere.00667-23-s0004.xlsx BGCs in the three strains' genomes.

Table S4 msphere.00667-23-s0005.xlsx BGCs and recovered metabolites.

ASM does not own the copyrights to Supplemental Material that may be linked to, or accessed through, an article. The authors have granted ASM a non-exclusive, world-wide license to publish the Supplemental Material files. Please contact the corresponding author directly for reuse.
