# Supplementary material for: Oligomycin-producing Streptomyces sp. newly isolated from Swiss soils efficiently protect Arabidopsis thaliana against Botrytis cinerea
Source: mSphere. 2024 Jun 12;9(7):e00667-23. doi: 10.1128/msphere.00667-23 (PMC11288007; doi:10.1128/msphere.00667-23)

**Supplementary materials**

for the article

**Oligomycin-producing *Streptomyces* sp. newly isolated from Swiss soils efficiently protect *Arabidopsis thaliana* against *Botrytis cinerea***

by

Fanny Louviot, Ola Abdelrahman, Eliane Abou-Mansour, Floriane L’Haridon, Pierre-Marie Allard, Laurent Falquet and Laure Weisskopf

**Supplementary Figures S1- S9**

**Figure S1:** Volatile-mediated inhibition of *Botrytis cinerea* mycelial growth by *Streptomyces* strains S5.1, S11.8, and S13.2. (A) Pictures of the split plate assay. The *Streptomyces* strains were inoculated on Bennett medium, while the fungus was cultured on PDA medium, either as a 5 mm plug (light gray circles), or a 10 µl spore suspension (dark gray circles). (B) Bar chart showing *B. cinerea* mycelium growth when exposed to volatiles emitted by strains S5.1, S11.8, and S13.2, presented as a percentage of the control growth. Each bar represents the average of five replicates with standard deviation error bars. Asterisks represent statistically significant differences in comparison to the control (Student’s t-test; ***p < 0.001, **p < 0.01, and *p < 0.05).


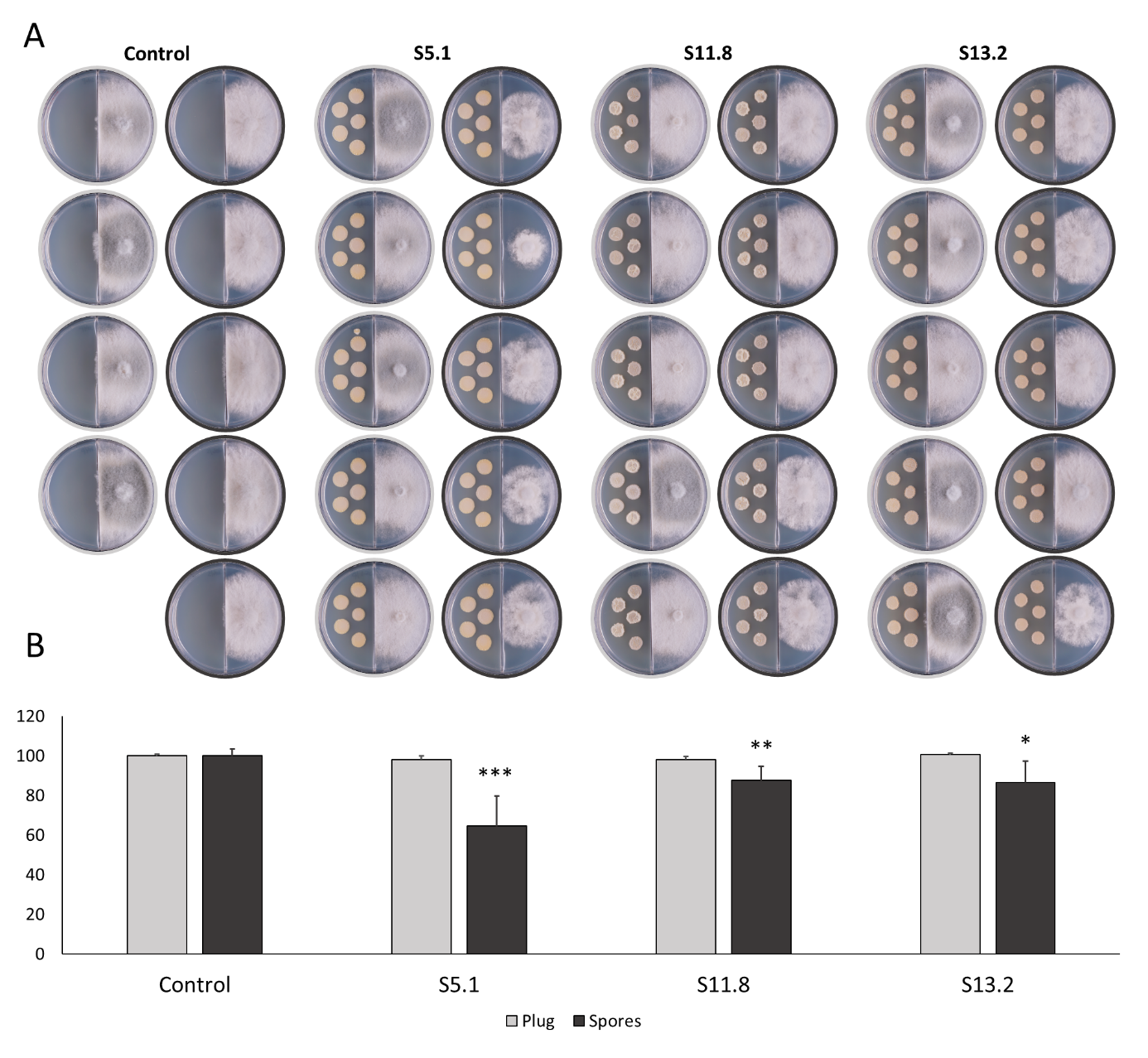


**Figure S2** High concentrations of CFF of the strains S11.8 and S13.2 are able to kill the spores of *Botrytis cinerea.* Spore germination of *Botrytis cinerea* incubated in media containing 10% of strain S11.8 and S13.2 CFF were visualized after eight days. Before taking the pictures, propidium iodide was added to the wells (see material and methods for more details). Pictures were taken with the Cytation 5 Cell Imaging Reader from Biotek at a 4-fold magnification.

**
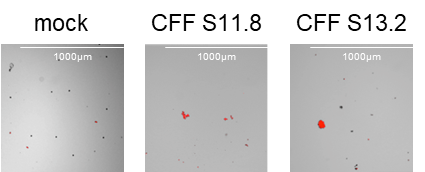
**

**Figure S3** HPLC analysis of the CFF from the strains S5.1, S11.8 and S13.2. A) Chromatogram, UV spectrum and mass spectra of germicidin A (RT 8.1) and B (RT 6.4) and of CFFs at 290 nm. B) Chromatogram, UV spectrum and mass spectra of oligomycin A (RT 25.3), B (RT 23.9) and E (RT 22.1) and of CFFs at 222 nm.


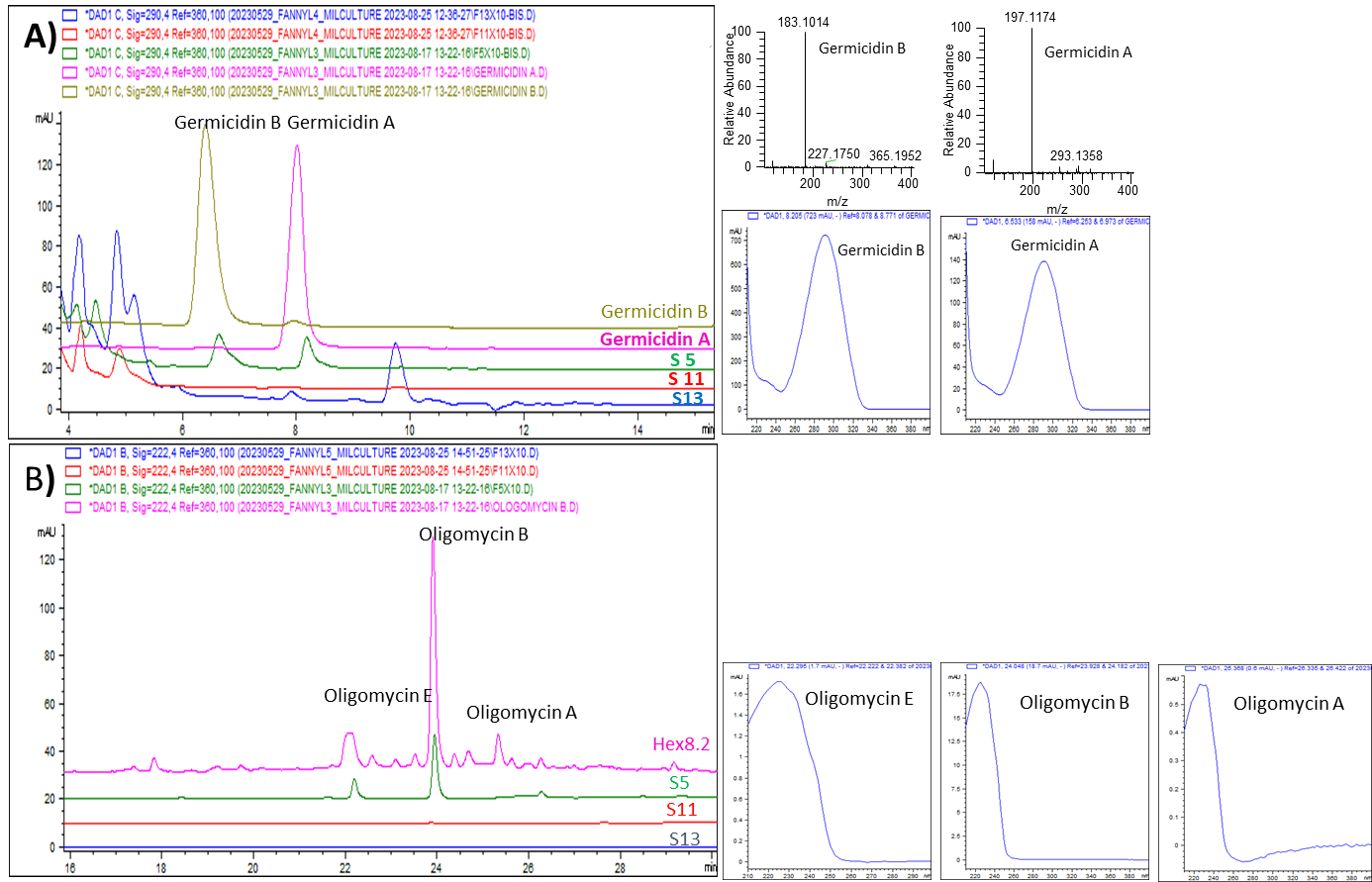


**Figure S4** Effect of hexane (HE), ethyl acetate (EAE) and methanol (ME) extracts of strain S5.1 against the mycelial growth of *B. cinerea* on PDA plates using the disc diffusion method. Extracts were dissolved in methanol and dried. The mycelial growth was observed at 72 h after incubation at 19°C with 12 hours of light/12 hours of darkness.


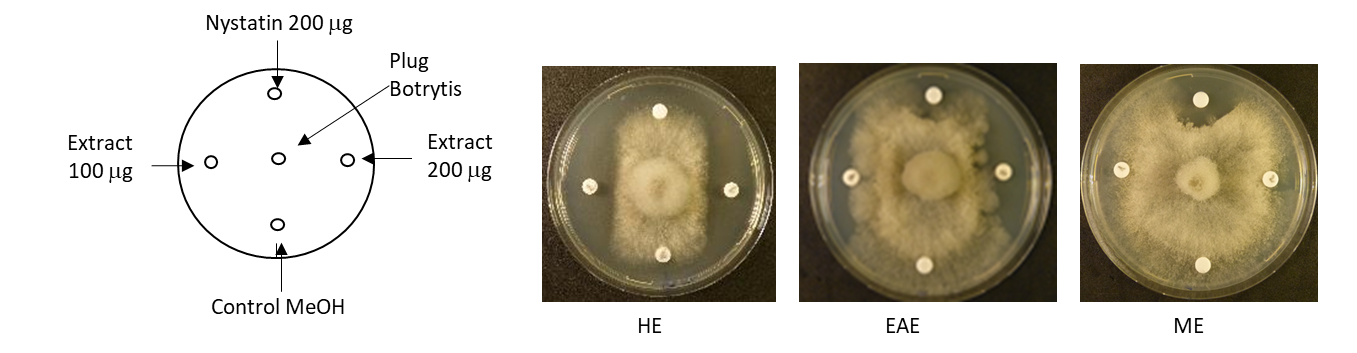


**Figure S5** MS/MS spectra of Oligomycin A (top), Oligomycin B /middle), and Oligomycin E (bottom)


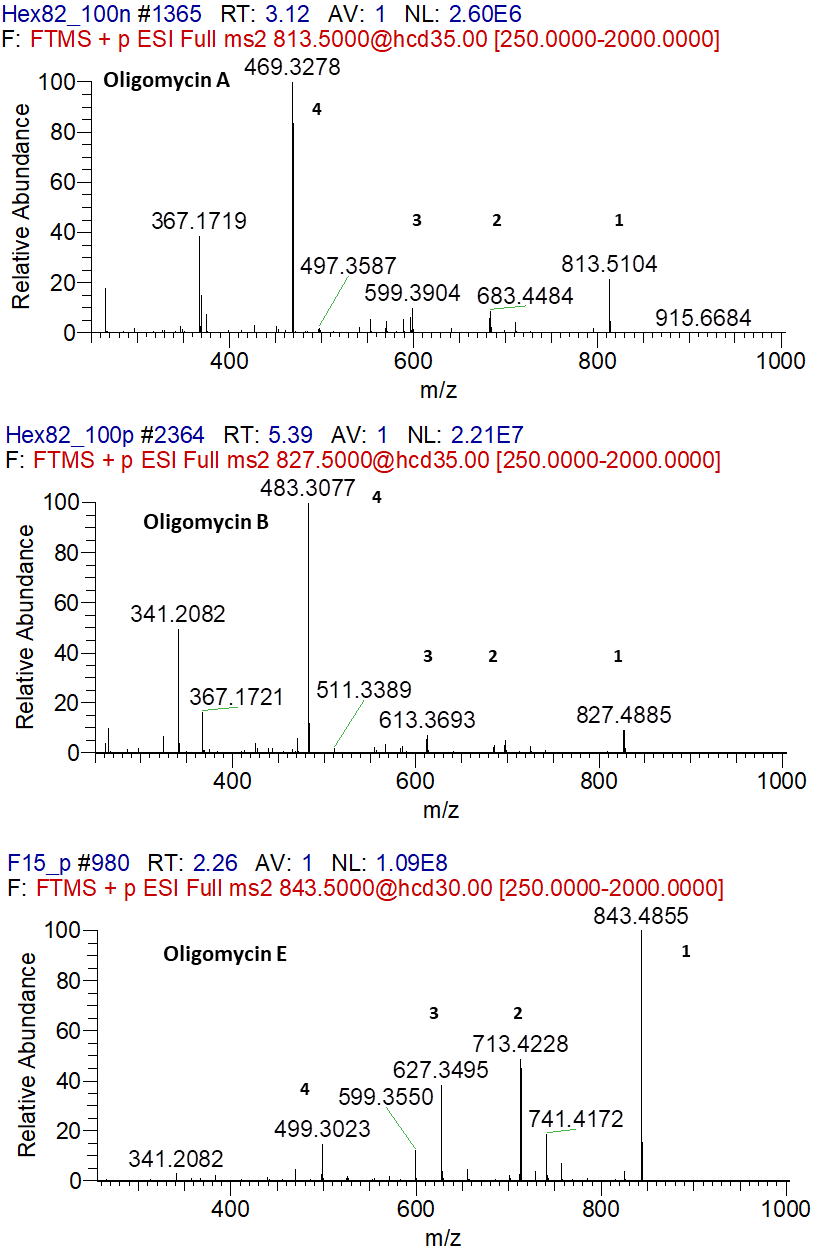


**Figure S6** ESI-MS/MS fragmentation of Oligomycin A, Oligomycin B, and Oligomycin E


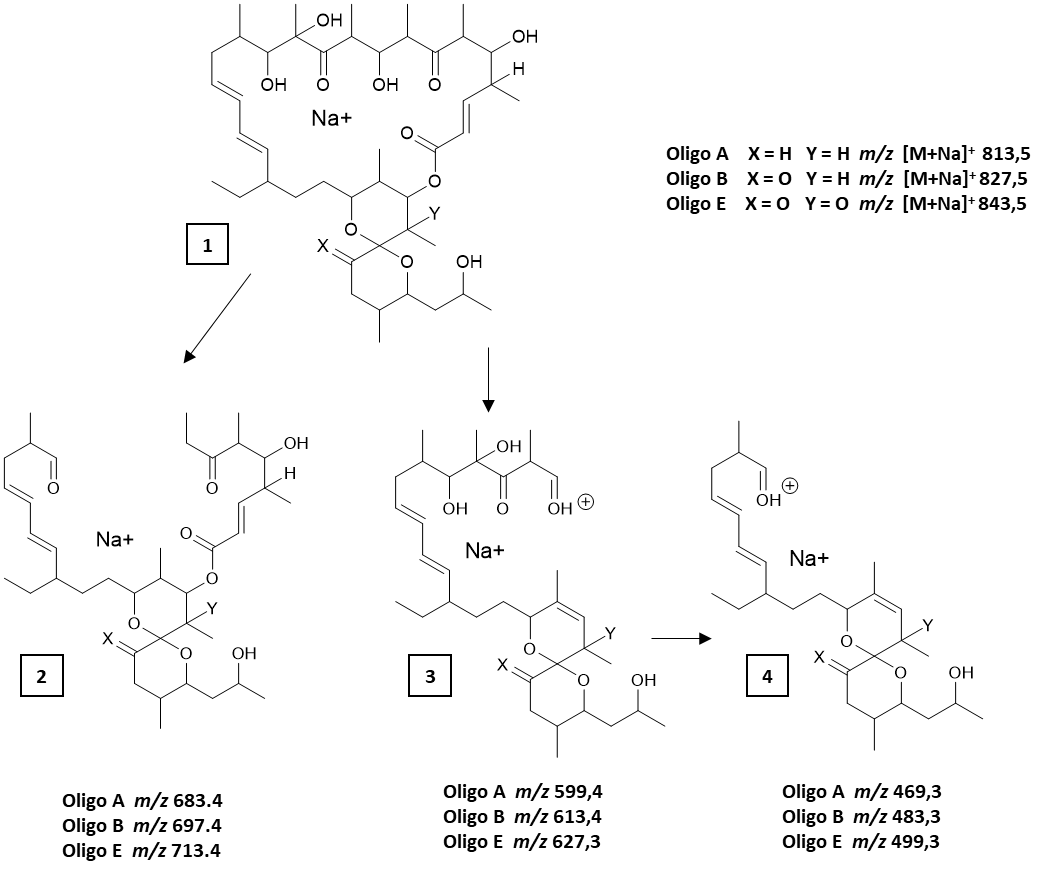


**Figure S7** Activity of germicidin A and B on *B. cinerea* mycelial growth. Effect of a fraction with equal mixture of germicidin A and B isolated from strain S5.1 against the mycelial growth of *B. cinerea* on PDA plates using the disc diffusion method. Compounds were dissolved in MeOH. Colony growth was observed at 72 h after incubation at 19 °C.


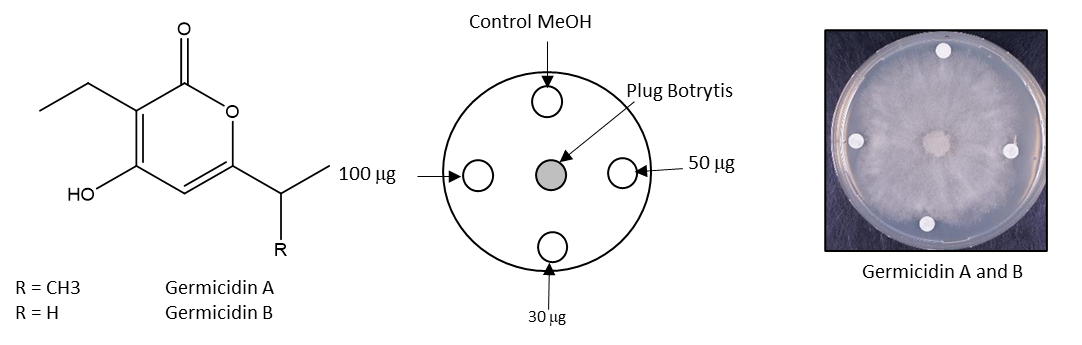


**Figure S8 Activity of purified germicidin A and B on the germination of *B. cinerea* spores.**

The effect of germicidin A and B on the germination of *B. cinerea* spores was assessed at different concentrations (20, 10 and 5 µg/mL). Each purified oligomycin was solubilized in ethanol to obtain a concentration of 0.6 mg/mL and thereafter diluted in PDB to obtain the three final concentrations tested. Ethanol diluted in PDB was the control. In each well, 198 µL of each solution was mixed with 2 µL of *B. cinerea* spores at a concentration of 1x10^6^ spores/mL. After an incubation of 24h at 20°C, pictures were taken with the Cytation 5 Cell Imaging Reader from Biotek at a 10-fold magnification. For each treatment, three replicates were analysed.


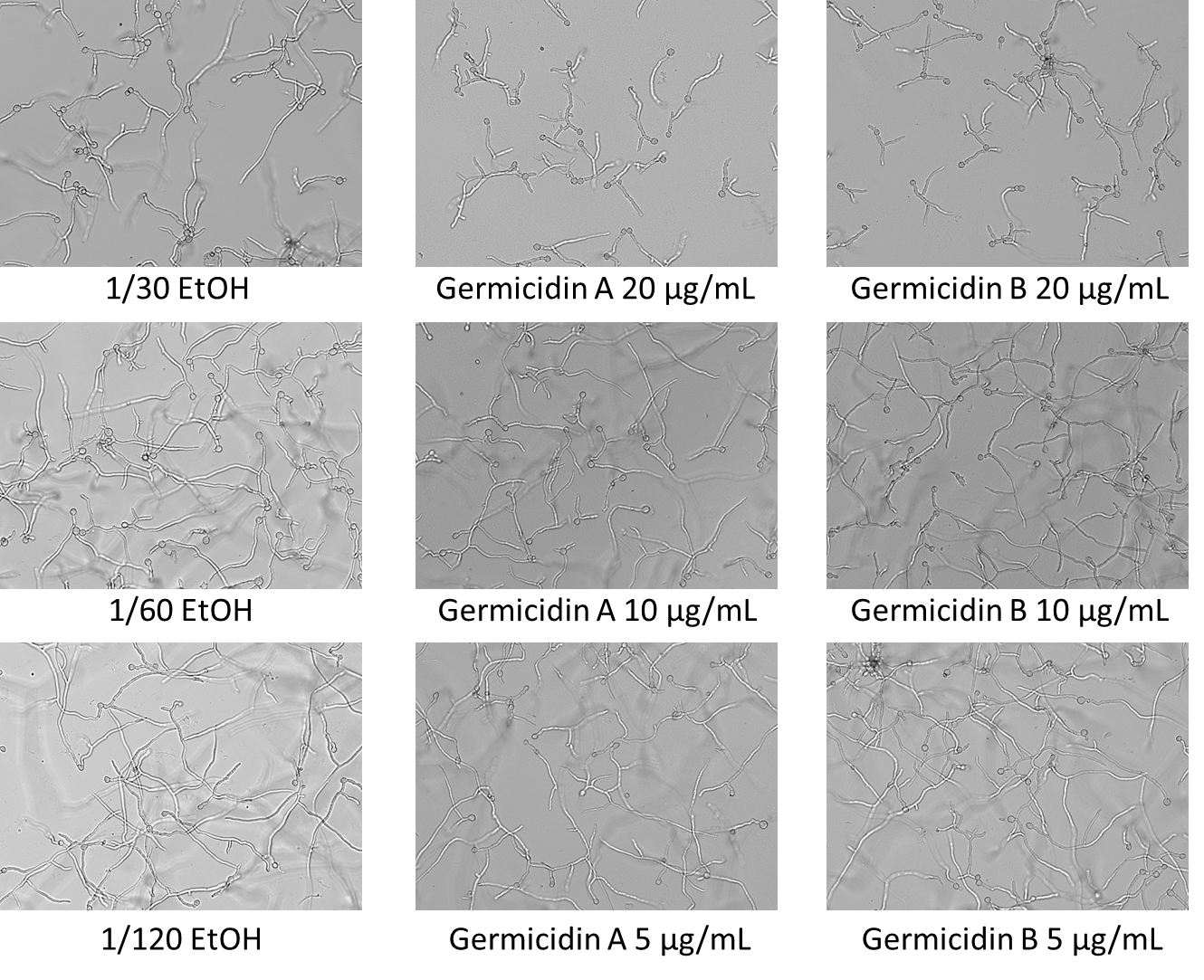


**Figure S9 Reduction of infection symptoms following soil inoculation with *Streptomyces* spores.** The soil in which *A. thaliana* plants were growing was inoculated with spores of either of the three *Streptomyces* strains (S5.1, S11.8 or S13.2) or treated with distilled water (mock). Left, the size of the lesions following *B. cinerea* infection in *A. thaliana* plants were measured three days post infection in two independent experiments (Exp 1 and Exp 2). Bars represent the averages of lesion sizes from 54 infected leaves from nine different plants separated in three independent groups. Statistical analyses indicated that there was no batch effect, allowing us to pool the data within each experiment. Significant differences between treatments according to a Student’s t test (P<0.05, n=54) are indicated by different letters. Right, representative pictures of infected leaves from plants grown in soil supplemented or not with *Streptomyces* spores. Arrows are pointing to the lesions.


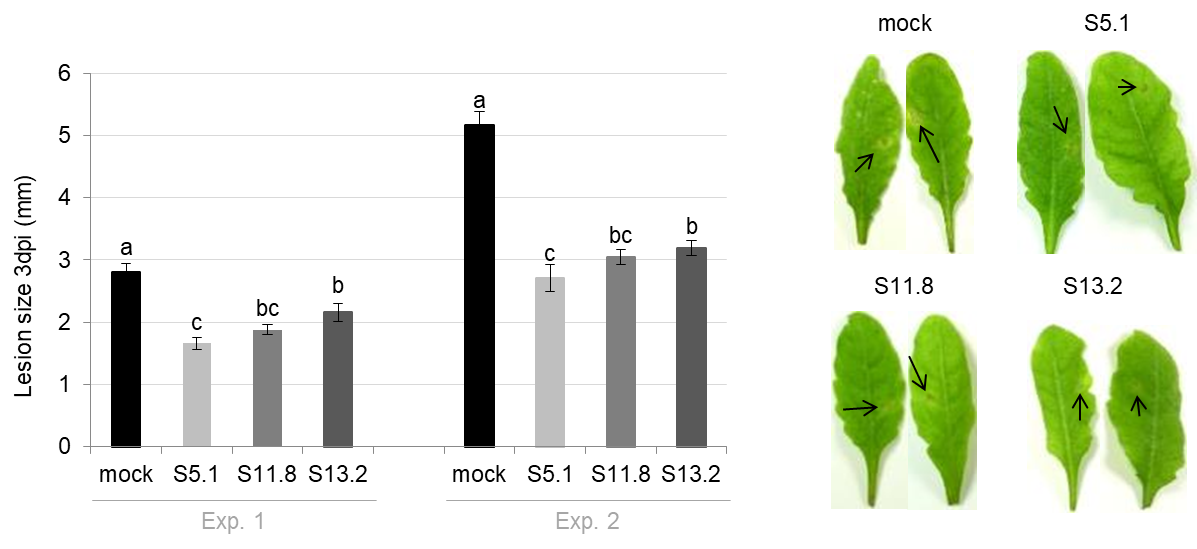

Supplement: Supplemental figures — Figures S1-S9. [file msphere.00667-23-s0001.docx]
